# Real world data on IO-based therapy for metastatic renal cell carcinoma

**DOI:** 10.1007/s00432-022-04173-0

**Published:** 2022-07-30

**Authors:** Viktoria Stühler, Lisa Herrmann, Steffen Rausch, Arnulf Stenzl, Jens Bedke

**Affiliations:** Department of Urology, University Hospital Tuebingen, Eberhard-Karls-University Tuebingen, Hoppe-Seyler Street 3, 72076 Tuebingen, Germany

**Keywords:** Immune checkpoint inhibitor, Immuno-oncology, Renal cell carcinoma, Tyrosine kinase inhibitor

## Abstract

**Purpose:**

Immune-based (IO)-combinations are the backbone in the systemic therapy of metastatic renal cell carcinoma (mRCC). Despite phase III clinical trial data, real world data are of special importance to reflect clinical practice.

**Methods:**

This retrospective study included 201 mRCC patients receiving first-line systemic therapy from January 2006. Clinicopathological and treatment-related data were recorded. Progression-free (PFS) and overall survival (OS) were analyzed using descriptive statistics and Kaplan–Meier analysis.

**Results:**

Over the years, IO-based therapies have increased significantly. The collective comprises 76 patients with first-line IO-based therapy (IO-IO:55, TKI-IO:21) and 125 patients with TKI-monotherapy. PFS was significantly improved with TKI-IO combinations if compared to both TKI-monotherapy (23.9 vs. 10.3 months, HR 0.48, *p* = 0.034) and IO-IO combination (23.9 vs. 6.1 months, HR 0.37, *p* = 0.012). OS for TKI-IO treated patients was longer compared to TKI-monotherapy (HR 0.37, p = 0.050) at median follow-up of 24.1 versus 29.9 months. In a subanalysis of nivolumab treated patients, starting from second-line (*n* = 40), PFS was 5.5 months. The addition of nivolumab either in second-or later lines improved OS compared to repeated TKI- or mTOR-therapies alone (6.13 vs. 2.61 years, HR 0.46, *p* = 0.003).

**Conclusion:**

Both first-line IO-based combinations and nivolumab after first-line TKI-monotherapy prolong OS in a real-world setting.

## Introduction

Worldwide, approximately 30% of new diagnosed renal cell carcinomas have advanced or metastatic disease (mRCC) with no curative treatment available (2015). In these cases, systemic therapy is the preferred treatment strategy and has evolved rapidly over the past few decades, with the increasing understanding of the biology and pathogenesis of the disease, incorporating targeted therapies such as vascular endothelial growth factor (VEGF)-directed tyrosine kinase inhibitors (TKIs) and immune-based (IO) therapies and resulting combinations. To date, there are different types of combination therapies for the first-line treatment depending on the International Metastatic Renal-Cell Carcinoma Database Consortium (IMDC) score: IO-IO combination with ipilimumab plus nivolumab in IMDC intermediate and poor-risk group, and four TKI-IO combinations of axitinib plus pembrolizumab, axitinib plus avelumab, cabozantinib plus nivolumab and lenvatinib plus pembrolizumab across all IMDC risk groups. As an alternative, TKI monotherapies with sunitinib, pazopanib, or cabozantinib, the latter agent only in the IMDC intermediate and poor-risk group can be used (Bedke et al. 2021). In two recently published meta-analysis from phase III randomized clinical trials with advanced or metastatic therapy-naïve RCC, the magnitude of benefit of TKI-IO combination versus sunitinib monotherapy was consistent across all clinicopathologic subgroups (Rizzo et al. 2021; Massari et al. 2021). With this plethora of first-line combination therapies, the choice of therapy becomes increasingly difficult as both prognostic models and direct comparative clinical trials of these different treatment options are lacking. Real world data may provide guidance here. However, patients treated in routine clinical practice can differ from patients in the real world setting as they tend to be older and have more comorbidities than patients enrolled in clinical trials, which highlights the importance to study these populations (Elting et al. 2006; Mailankody and Prasad 2017).

The aim of the current study was to assess IO-based and TKI-based treatments in terms of progression-free (PFS) and overall survival (OS) in real-world academic setting and to analyze treatment change over time. The evidence derived from guidelines and recommendations is primarily from randomized clinical trials and thus on a more specific patient population.

## Material and methods

This single center retrospective study included 201 patients with mRCC of any histology receiving first-line systemic therapy as of January 2006 till March 2022 at the Department of Urology of the University of Tuebingen, Tuebingen, Germany. Before starting data collection, approval from the local research ethics committee was obtained (078/2012/B02). Clinical, pathological, and treatment-related parameters were recorded. Data collected included the time of primary surgery, TNM stage, grading, histological subtype, Karnofsky performance status, number of distinct metastases, first-line and subsequent lines of therapies. The Memorial Sloan-Kettering Cancer Center (MSKCC) risk score was calculated at the time of first metastasis. PFS was investigator assessed by axial imaging in intervals of approximately 12–16 weeks and of clinical progression. PFS and OS were analyzed using descriptive statistics and Kaplan–Meier curves, with follow-up calculated from the start of first-line systemic therapy until death from any cause or censored at the time of last follow-up. For multivariate analysis, clinically important parameters were included in addition to those significant in univariate analyses. Statistical analyses were performed using SPSS, version 27. A *p* < 0.05 was considered as a statistical significant difference.

## Results

### Patient characteristics

**Fig. 1:**
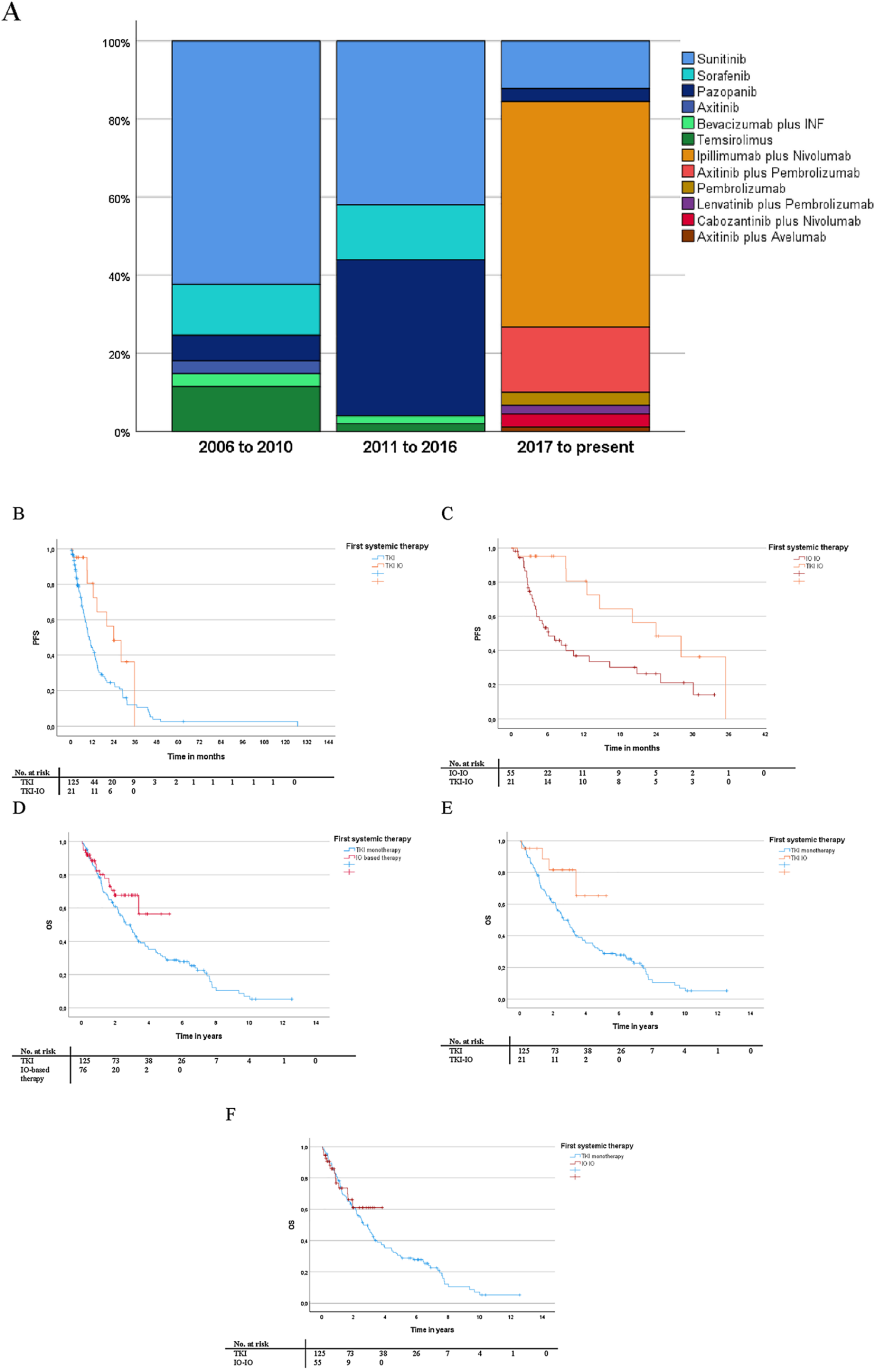
**A** Sequential treatment strategies over time in patients with mRCC. Percentages may not add to 100% due to rounding. **B-C** Kaplan–Meier analyses for PFS depending on first-line systemic therapy with TKI-IO vs. TKI monotherapy (**B**) and vs. IO-IO combination (**C**). PFS defined as time from first systemic therapy to tumor progression. **D-F** Kaplan–Meier analyses for OS depending on first-line systemic therapy with IO-based therapy vs. TKI monotherapy (**D**), TKI monotherapy vs. TKI-IO (**E**) or vs. IO-IO (**F**). OS defined as time from start first systemic therapy to death/last follow up

Over the past 15 years, the number of IO-based systemic therapies has increased remarkably. As of 2017, 57.8% of patients received ipilimumab plus nivolumab, 26.7% a TKI-IO combination, and only 15.5% were treated with TKI monotherapy in first-line. This change over time is presented in Fig. 1A. In total, the collective included 76 patients with first-line IO-based therapy, with 55 patients treated with an IO-(IO), 21 with a TKI-IO combination, and 125 patients with TKI monotherapy. IO-based therapies used were combinations of ipilimumab plus nivolumab (*n* = 52), pembrolizumab monotherapy (*n* = 3), axitinib plus pembrolizumab (*n* = 15) or avelumab (*n* = 1), lenvatinib plus pembrolizumab (*n* = 2), and cabozantinib plus nivolumab (*n* = 3). Patient demographics and baseline characteristics are summarized in Table 1.

**Table 1:**
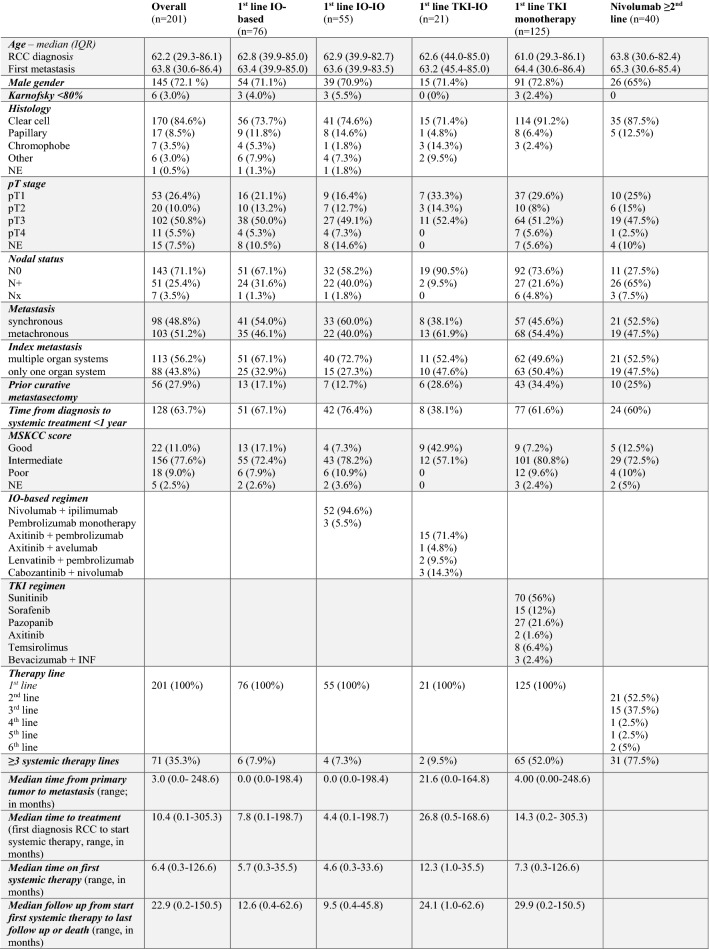
Baseline characteristic of the total study cohort and in the subgroup of patients with first-line IO-based therapy, IO-IO and TKI-IO combinations, TKI monotherapy, and Nivolumab ≥ second-line

*G* grading, *IQR* interquartile range, *IO* immuno-oncology, *M* distant metastasis, *MSKCC* memorial sloan-kettering cancer center (motzer) score, *N* regional lymph nodes, *NE* not evaluable, *PFS* progression-free survival, *R* resection status, *RCC* renal cell carcinoma, *T* primary tumor, *TKI* tyrosine kinase inhibitor.

Median time to treatment (MTT) from initial diagnosis to start of systemic therapy was 10.4 months for the overall population with 14.3 months for the TKI monotherapy, and 7.8 months for the IO-based subgroup. Here, MTT was 4.4 months for IO-IO and 26.8 months for TKI-IO groups. Median follow-up time from the start of first systemic therapy was 12.6 months for IO-based therapy (range, 0.4–62.6 months; IO-IO: 9.5 months, TKI-IO: 24.1 months) and 29.9 months for TKI monotherapy (range, 0.2–150.5 months). Prior first-line metastasectomy was performed in 56 patients (27.6%), with 13 patients treated with an IO-based approach at recurrence and 43 patients receiving TKI monotherapy. Tabulation of patients according to MSKCC criteria shows that 13 patients (17.1%) who received IO-based therapy were classified in the favorable-risk group, with 9 patients treated with a TKI-IO and 4 patients treated with an IO-IO combination. Only 9 patients who received TKI monotherapy were in the MSKCC favorable-risk group. Patients with first-line IO-based therapy were more likely to have synchronous disease (54.0 vs. 45.6%) and multiple organ systems were affected (67.1 vs. 49.6%).

###  Survival analysis

Median time on first-line systemic therapy was 6.4 months for the overall population with 7.3 months for patients treated with a TKI monotherapy compared to 5.7 months for an IO-based approach, with 4.6 months for the IO-IO and 12.3 months for the TKI-IO subgroups. In terms of PFS, first-line TKI-IO combination significantly improved PFS compared to both TKI monotherapy (23.9 vs. 10.3 months, HR 0.48, 95% CI 0.24–0.95, *p* = 0.034) and IO-IO combination (23.9 vs. 6.1 months, HR 0.37, 95% CI 0.17–0.80, *p* = 0.012). Other clinical parameters such as timing of metastasis (synchronous vs. metachronous), time between nephrectomy and metastasis < 1 year, number of index metastases, and the MSKCC risk score did not significantly affect PFS. The results of univariate analyses are summarized in Table 2A with the corresponding Kaplan–Meier curves in Fig. 1B and C. Multivariate analysis confirmed a significantly improved PFS for TKI-IO versus IO-IO combination (HR 0.37, 95% CI 0.16–0.87, *p* = 0.023) and an improved PFS of TKI-IO if compared to TKI monotherapy (HR 0.48, 95%CI 0.23–1.02, *p* = 0.057), see Table 2D.

**Table 2:**
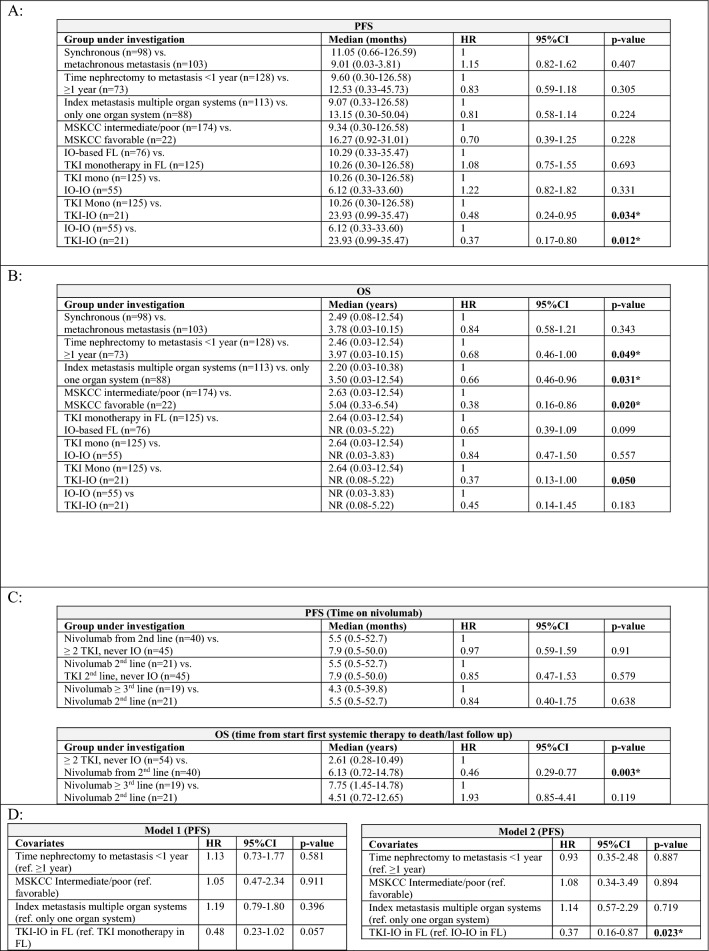

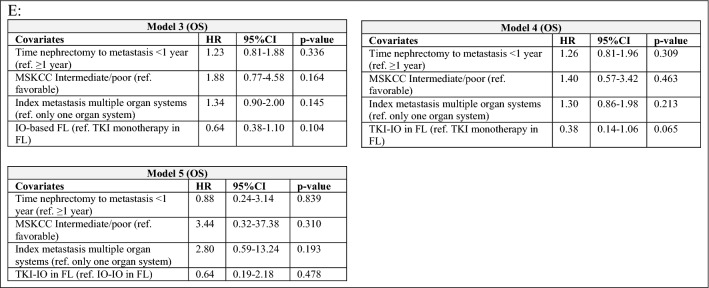
A Overview of calculated PFS depending on clinical parameters as well as first-line therapies and univariate analyses of PFS, defined as time from first systemic therapy to progression. B Univariate analysis of OS, defined as time from start first systemic therapy to death/last follow-up. C Overview of calculated PFS and OS depending on nivolumab beyond first-line setting. D-E Multivariate analyses of PFS and OS for clinical parameters

*FL* first-line therapy, *IO* immuno-oncology, *MSKCC* memorial sloan-kettering cancer center (motzer) score, *NR* not achieved, *PFS* progression-free survival, *Ref*. reference, *OS* overall survival, *TKI* tyrosine kinase inhibitor.

Median OS was not yet reached for IO treated patients, with an advantage over TKI monotherapy (NR vs. 2.64 years, HR 0.65, 95% CI 0.39–1.09, *p* = 0.099). OS was longer with the TKI-IO combination than with TKI monotherapy (NR vs. 2.64 years, HR 0.37, 95% CI 0.13–1.0, *p* = 0.050). Here, median OS was not yet reached for both first-line IO-IO and TKI-IO combination therapy. However, a numerical advantage was observed for the TKI-IO combination over the IO-IO combination (HR 0.45, *p* = 0.183).

Other clinical parameters significantly associated with OS included time between nephrectomy and metastasis < 1 year, MSKCC risk score, and the number of organ systems affected at the time of first metastasis. Thus, the median OS was 5.04 years in patients with favorable versus 2.63 years in MSKCC patients with intermediate/poor risk (HR 0.38, 95% CI 0.16–0.86, *p* = 0.020). Moreover, metastasis to only one organ system at the time of first metastasis was a better prognostic factor for OS compared to metastasis to at least two organ systems (3.5 vs. 2.2 years, HR 0.66, 95% CI 0.46–0.96, *p* = 0.031). However, multivariate analysis confirmed only a non-significant improvement in OS for the first-line TKI-IO combination compared to TKI monotherapy (HR 0.38, *p* = 0.065).

### Nivolumab subgroup

In a subanalysis of nivolumab after first-line therapy (*n* = 40), 21 patients received nivolumab in the second-line setting and 19 patients received nivolumab starting in the third-line setting (3rd line *n* = 15, 4th line *n* = 1, 5th line *n* = 1, 6th line *n* = 2). All patients had been previously treated with TKI monotherapies or mTOR inhibitors only. PFS was 5.5 months for both the total nivolumab collective (*n* = 40) and second-line nivolumab (*n* = 21), while PFS for nivolumab ≥ third-line was 4.3 months (*n* = 19). There was no significant difference in PFS between nivolumab from second-line and second-line TKI monotherapy (*p* = 0.91 and *p* = 0.579, respectively). The addition of nivolumab either in the second-line or beyond significantly improved OS calculated from the start of first systemic therapy compared to repeated TKI or mTOR therapies alone (≥ 2 TKIs; median 6.13 vs. 2.61 years, HR 0.46, 95% CI 0.29–0.77, *p* = 0.003).

## Discussion

Over the past two decades, systemic therapy for mRCC has undergone several revolutions, first with the introduction of targeted therapies such as TKIs, later with antibodies targeting the programmed death receptor axis (PD-1, IO therapies), and finally by combining them (Escudier 2019). The aim of the current study was to investigate the systemic therapies, their use over time and to assess PFS and OS rates in the real world setting of an academic institution. A retrospective study cohort of 201 mRCC patients treated with either IO-IO, TKI-IO combinations, or TKI monotherapy at our reference center is presented, starting in January 2006.

First, our heterogeneous cohort of patients treated with a variety of therapies over the past 2 decades reflects the current mRCC reality. It is evident that newly approved agents have been rapidly implemented into routine practice. As of 2017, 57.8% of patients have received ipilimumab plus nivolumab, 26.7% a TKI-IO based combination, and only 15.5% are still on TKI monotherapy for first-line mRCC treatment. An analysis of the 2018 German RCC clinical registry showed the rapid implementation of IO therapy with only 1% of patients treated with first-line IO-based therapy between 2015 and 2017, whereas nearly 20% received second-line IO-based therapy which was recently approved by that time (Goebell et al. 2018). With the plethora of first-line combination therapies, the choice of therapy is becoming increasingly difficult. As randomized phase III clinical trials directly comparing the spectrum of new first-line options are unlikely to be conducted, real-world data will be critical to guide clinical, policy, and financial decisions. However, patients in routine practice differ from patients treated in clinical trials primarily by lower performance status and a higher number of impairing comorbidities (Marschner et al. 2017; Mitchell et al. 2015; Heng et al. 2014).

Second, we showed that investigator assessed PFS was significantly improved for first-line TKI-IO combination compared to TKI monotherapy as well as versus IO-IO combination (*p* = 0.034, *p* = 0.012). Multivariate analysis confirmed the PFS benefit of TKI-IO versus IO-IO combination (*p* = 0.023). The PFS of 23.9 months with TKI-IO combinations after a median follow-up of 24.1 months obtained in our study compared favorably with that observed in phase III clinical trials. To date, all phase III clinical trials of TKI-IO combinations have demonstrated improved PFS over comparator sunitinib across all IMDC risk groups, for axitinib plus pembrolizumab (Keynote 426: 15.7 months with median follow-up 42.8 months, HR 0.68, *p* < 0.0001), for axitinib plus avelumab (Javelin renal 101: 13.8 months (PD-L1 + subgroup) with median follow-up 19 months, *p* < 0.0001), for cabozantinib plus nivolumab (CheckMate 9ER: 17.0 months with median follow-up 23.5 months, HR 0.52, *p* < 0.0001), and for lenvatinib plus pembrolizumab (CLEAR: 23.9 months with median follow-up 33.4 months, *p* < 0.001) (Powles et al. 2020, Rini et al. 2021, Choueiri et al. 2020, Choueiri et al. 2021, Motzer, Choueiri, et al. 2021, Motzer et al. 2021a, b, Choueiri T 2021). The PFS of 6.1 months for ipilimumab plus nivolumab observed in our cohort is much shorter as the PFS observed in the Checkmate 214 trial (11.2 months) (Albiges et al. 2020). This could be on the one hand related to either the investigator assessment and not a blinded independent review as done in phase III clinical trials, on the other a different risk group balance between our real-world cohort and the clinical trial setting. In addition, a recent retrospective, multi-institutional cohort analysis also demonstrated a shorter median PFS after initiation of IO-based treatment of 9.8 months (Hoeh et al. 2022).

Third, in our cohort, a numerical improvement in OS was observed with first-line IO-based therapy over TKI monotherapy (NR vs. 2.64 years, HR 0.65, *p* = 0.099). The lack of significant difference may be due to the difference in follow-up time between the study groups: 1.1 years for the IO-based group versus 2.5 years for the TKI monotherapy group. For the same reason, OS was numerically improved for the TKI-IO combination compared with TKI monotherapy, but failed to reach the threshold of statistical significance (NR vs. 2.64 years, HR 0.37, 95% CI 0.13–1.0, *p* = 0.050). Median OS was not achieved for first-line therapy with IO-IO or TKI-IO combinations, and although there was a benefit for the TKI-IO combination with a HR of 0.45, this is likely related to the short follow-up period (*p* = 0.183). Furthermore, exploratory multivariable analysis confirmed a trend toward better OS for first-line TKI-IO combination compared to TKI monotherapy (HR 0.38, *p* = 0.065). Regarding phase III clinical trial results, TKI-IO combinations lead to an OS benefit compared to sunitinib with in Keynote-426 median 45.7 months (ITT population, median follow-up 42.8 months, HR 0.73, *p* = 0.001); in CheckMate 9ER not achieved (ITT population, median follow-up 23.5 months, HR 0.66, *p* = 0.0034); and in CLEAR not achieved (ITT population, median follow-up 33.4 months, HR 0.72, *p* = 0.005) (Powles et al. 2020; Rini et al. 2021; Motzer et al. 2021a, b; Choueiri T 2021; Choueiri et al. 2021; Motzer, Choueiri et al. 2021). For axitinib plus avelumab, there is still no significant impact on OS at a median follow-up of 19 months in the Javelin Renal 101 trial (NR, HR 0.83, *p* = 0.1301) (Motzer et al. 2019; Choueiri et al. 2020).

Our analysis points out that the OS benefit of TKI-IO combinations as observed in clinical trial cohorts also applies to this cohort of patients treated in real-world clinical practice, confirming the applicability of these findings on a broader basis. However, we were unable to confirm the significant improvement of median OS of 48.1 months (HR 0.65, *p* < 0.0001) observed in the Checkmate 214 trial of ipilimumab plus nivolumab in intermediate/low-risk IMDC patients in our cohort (HR 0.84, *p* = 0.557), so far (Albiges et al. 2020).

Regarding differences in OS within IO-based therapies, our data show that median OS for first-line IO-IO or TKI-IO combinations is not yet mature and needs long follow time to draw definitive conclusions. Although a non-significant improvement was demonstrated for the TKI-IO combination with a HR of 0.45 (*p* = 0.183), interpretation should be made with caution due to the limited number of events in the IO-based group. Our results are similar to findings from recently published studies. In a retrospective cohort study from the National Cancer Database, it was found that in patients with clear cell mRCC, both IO-IO and TKI-IO combinations were associated with significantly better OS than TKI monotherapy (for IO-IO group: HR 0.60, p < 0.001; for the TKI-IO combination group: HR 0.74, *p* = 0.005), with no survival difference observed between the TKI-IO and IO-IO combination (HR 1.24, 95% CI, 0.98–1.56, *p* = 0.08) (Chakiryan et al. 2021). Moreover, in the aforementioned retrospective multi-institutional cohort analysis with real-world data, OS rates were comparable between first-line IO-IO and TKI-IO treatment approaches, with OS rates at 12 months of 73.9 versus 90.0% (*p* = 0.089), respectively (Hoeh et al. 2022). Results from the International Metastatic Renal-cell Carcinoma Database Consortium (*n* = 113 for TKI-IO combinations, *n* = 75 for ipilimumab plus nivolumab) also confirmed the non-significant differences in OS between first-line IO-IO and TKI-IO treatment (median OS NR vs. NR, after adjustment for IMDC risk factors *p* = 0.14) (Dudani et al. 2019).

Forth, given the routine use of nivolumab in mRCC patients from second-line onward, we also provide a qualitative assessment of the efficacy of this IO monotherapy after first-line in our cohort. Our collective reflects past reality as all patients received prior TKI monotherapies or mTOR inhibitors followed by nivolumab from second-line. Our data show a median PFS of 5.5 months for second-line treatment with nivolumab compared to 7.9 months for second-line treatment with TKI monotherapy (*p* = 0.91). The addition of nivolumab either in the second-line or beyond significantly improved OS compared to a TKI or mTOR therapy alone (≥ 2 TKIs; median 6.13 vs. 2.61 years, HR 0.46, *p* = 0.003). Regarding the associated pivotal study, the 5 year analysis of CheckMate 025 comparing nivolumab to everolimus in mRCC patients previously treated with 1–2 antiangiogenic therapies confirmed the superior efficacy of nivolumab over everolimus. At a minimum follow-up of 64 months (median 72 months), PFS favored nivolumab with 4.2 months (HR 0.84, *p* = 0.0331). In addition, nivolumab maintained an OS benefit over everolimus (median 25.8 vs. 19.7 months; HR 0.73, *p* < 0.0001), with 5 year OS probabilities of 26 and 18%, respectively. Both PFS and OS in our analysis were comparable to those in the Checkmate 025 trial, although patients in our analysis received a second-line TKI rather than the mTOR inhibitor everolimus as in the Checkmate 025 trial (Motzer et al. 2020). Real-world data on the use of nivolumab in 228 mRCC patients from the non-interventional NORA study showed that efficacy and safety in this real-world population were consistent with the pivotal clinical trial after a median follow-up of 37 months, with median PFS of 5.3 months and OS of 24 months (Grimm et al. 2021). These findings support the use of nivolumab after prior TKI monotherapy in mRCC patients.

Our analysis has several limitations. There is an inherent risk of selection bias in retrospective comparative effectiveness studies. In addition, sample size is limited and follow-up time, especially for the IO-based therapies, is variable and in some cases limited. Consequently, clinically meaningful differences for the end points in the IO combination groups cannot be excluded by the present analysis. Further reports with more mature data are warranted. Other important limitations include the lack of collecting toxicity data and response rates, which can only be indirectly measured by the PFS time interval from the start of systemic therapy. With regard to PFS, it should also be mentioned that tumor assessment in routine care was partly not performed according to the Response Evaluation Criteria In Solid Tumors (RECIST) used in clinical trials.

Another potential selection bias may occur as no standardized protocol was used for decision making regarding treatment regimens. However, this may make these data more reflective of real-world experience. Overall, the IO-based and TKI monotherapy cohorts are quite homogeneous in several patient and tumor characteristics, but there are still some differences.

Finally, the novelty of the study is limited, as numerous similar and larger studies have been published in recent years. Nevertheless, the data have value as they reflect the reality of the rapidly evolving therapeutic landscape for mRCC at a major urologic center and provide comparable results to pivotal trials.

## Conclusions

Our analysis shows evidence for an advantage of first-line IO-based combinations over TKI monotherapy emerging with longer follow-up of mRCC patients in real-world settings. Nivolumab from second line onward is also effective after TKI monotherapy outside of clinical trials.
